# Supplementary material for: Topoisomerase 1 cleavage complex enables pattern recognition and inflammation during senescence
Source: Nat Commun. 2020 Feb 19;11:908. doi: 10.1038/s41467-020-14652-y (PMC7031389; doi:10.1038/s41467-020-14652-y)
Supplement: Supplementary file 1 — Supplementary Information [file 41467_2020_14652_MOESM1_ESM.pdf]

Supplementary Information

Topoisomerase 1 cleavage complex enables pattern recognition and inflammation during senescence

Zhao et al.

Supplementary Figures

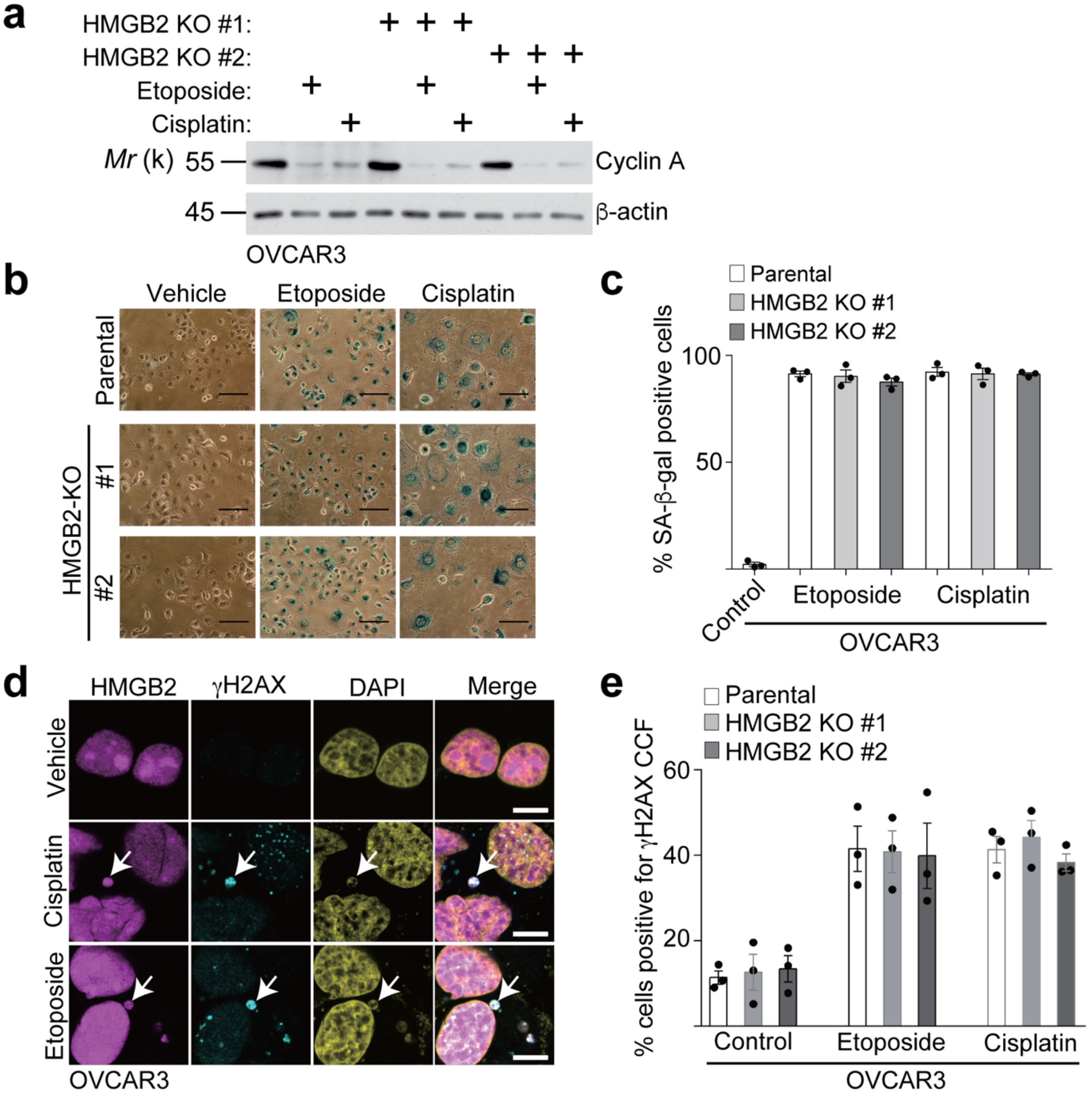

Supplementary Figure 1: HMGB2 is not required for formation of γH2AX positive CCF during therapy-induced senescence of ovarian cancer cells.

a, Parental and HMGB2 knockout OVCAR3 ovarian cancer cells were treated with Etoposide or Cisplatin to induce senescence. Expression of Cyclin A and a loading control β-actin in the indicated cells was examined by immunoblot. **b-c,** Representative images (**b**) and quantification (**c**) of SA-β-Gal staining of OVCAR3 cells with the indicated treatments. **d,** Co-staining of HMGB2 and γH2AX in control and senescent OVCAR3 cells induced by in the indicated treatments. Arrows point to CCF. **e,** Quantification of γH2AX positive CCF formation in the indicated control and therapy-induced senescent OVCAR3 cells. Data represent mean ± s.e.m. n = 3 biologically independent experiments. Scale bar = 100 μm in 1b. Scale bar = 10 μm in 1d. Source data are provided as a Source Data file.

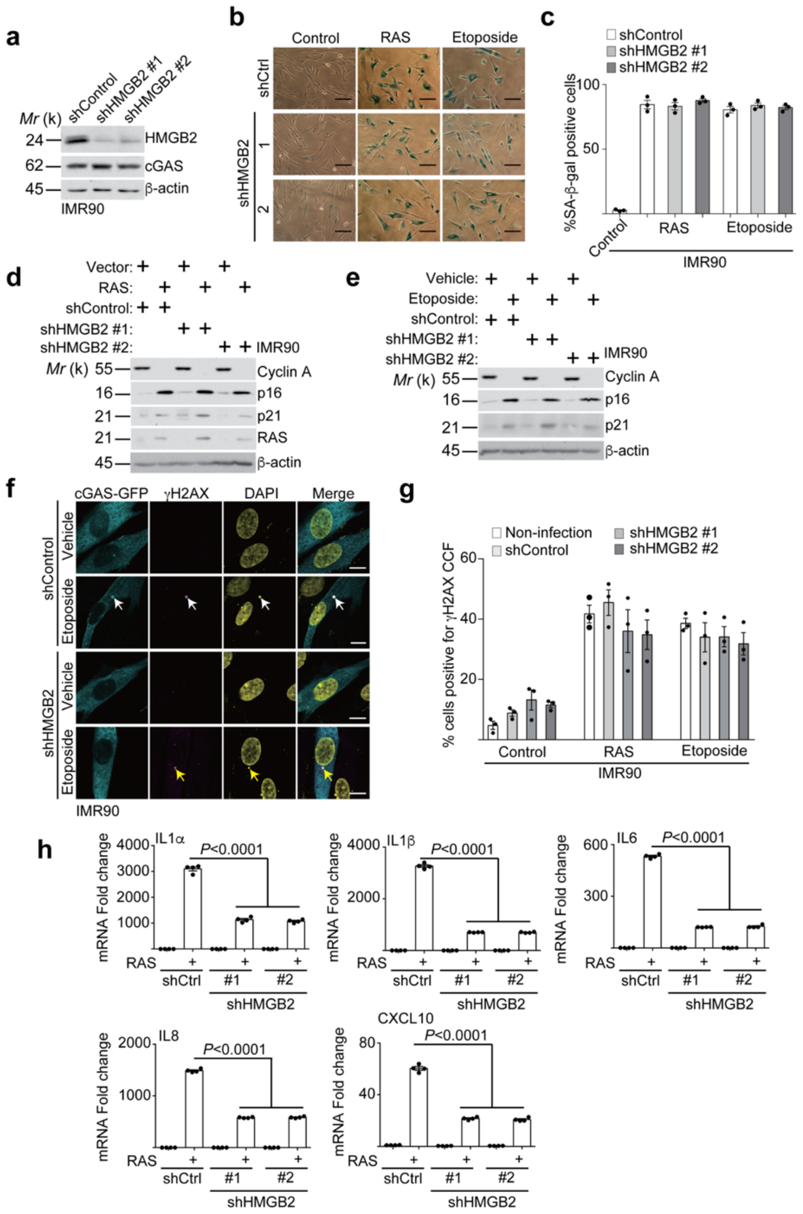

Supplementary Figure 2: HMGB2 is required for cGAS' localization into CCF.
a, Expression of HMGB2, cGAS and a loading control β-actin in IMR90 cells expressing the indicated shHMGB2s or control was determined by immunoblot. **b-c,** Representative images (**b**) and quantification (**c**) of SA-β-Gal staining in IMR90 cells expressing oncogenic RAS or treated with Etoposide to induce senescence (n = 3 biologically independent experiments). **d-e,** Expression of the indicated proteins in IMR90 cells induced to senesce by oncogenic RAS (**d**) or Etoposide (**e**) with or without HMGB2 knockdown was determined by immunoblot. **f,** cGAS-GFP localization into γH2AX positive CCF in Etoposide-induced senescent IMR90 cells with or without HMGB2 knockdown. Arrows point to CCF. **g,** Quantification of γH2AX positive CCF formation in the indicated control and RAS-induced senescent IMR90 cells (n = 3 biologically independent experiments). **h,** Expression of the indicated SASP factors in the indicated IMR90 cells determined by qRT-PCR (n = 4 biologically independent experiments). Data represent mean ± s.e.m. Scale bar = 100μm in 2b. Scale bar = 10μm in 2f. *P* values were calculated using a two-tailed *t*-test. Source data are provided as a Source Data file.

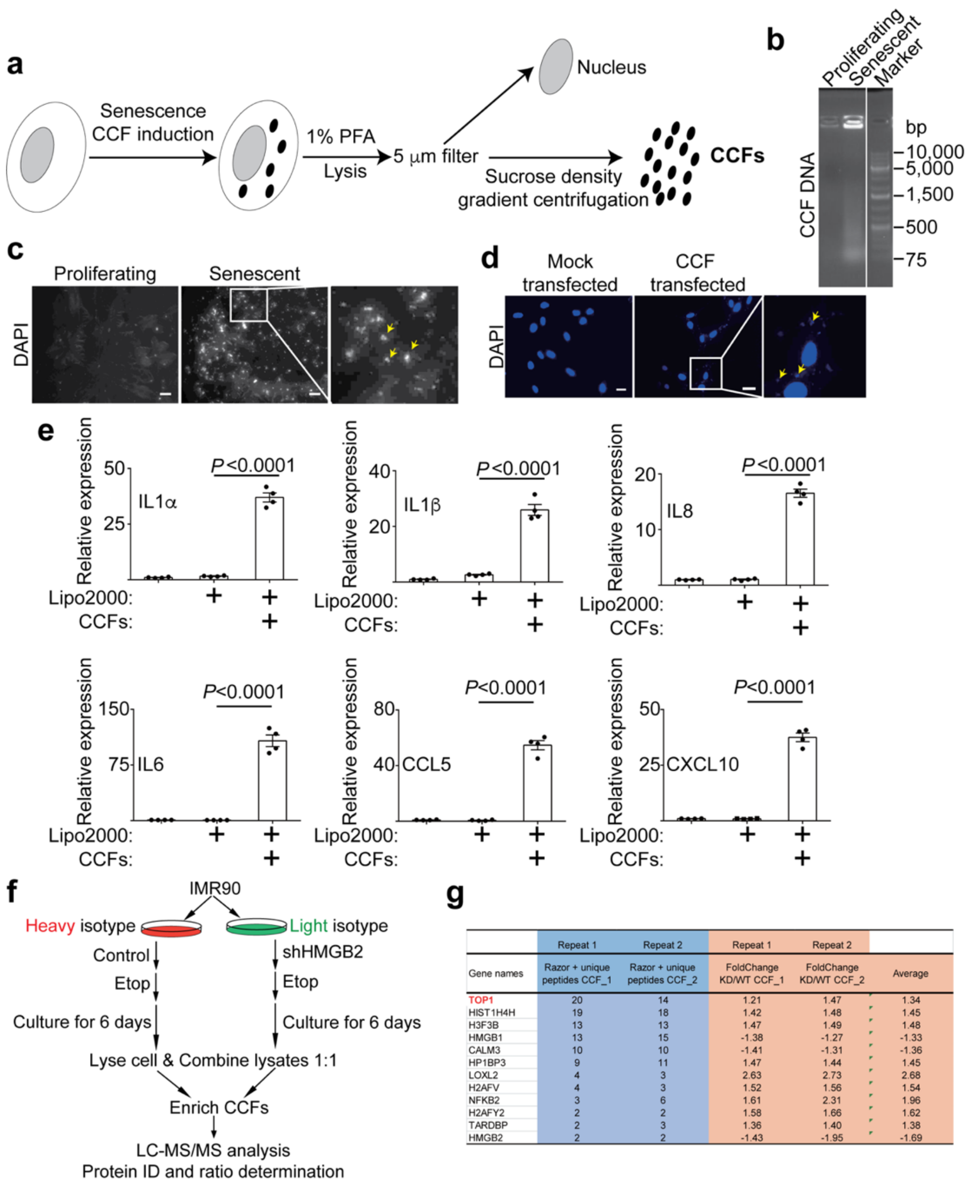

	Repeat 1	Repeat 2	Repeat 1	Repeat 2	
Gene names	Razor + unique peptides CCF_1	Razor + unique peptides CCF_2	FoldChange KD/WT CCF_1	FoldChange KD/WT CCF_2	Average
TOP1	20	14	1.21	1.47	1.34
HIST1H4H	19	18	1.42	1.48	1.45
H3F3B	13	13	1.47	1.49	1.48
HMGB1	13	15	-1.38	-1.27	-1.33
CALM3	10	10	-1.41	-1.31	-1.36
HP1BP3	9	11	1.47	1.44	1.45
LOXL2	4	3	2.63	2.73	2.68
H2AFV	4	3	1.52	1.56	1.54
NFKB2	3	6	1.61	2.31	1.96
H2AFY2	2	2	1.58	1.66	1.62
TARDBP	2	3	1.36	1.40	1.38
HMGB2	2	2	-1.43	-1.95	-1.69

Supplementary Figure 3: HMGB2 inhibition increases TOP1 levels in CCF.
a, Schematics of the protocol used for CCF purification. **b,** Agarose gel electrophoresis of DNA isolated from the purified CCF in senescent IMR90 cells induced by Etoposide. **c,** Purified CCF from Etoposide-induced senescent IMR90 cells visualized by DAPI staining. **d,** Purified CCF were transfected into IMR90 cells and the transfected cells were stained with DAPI to visualize the transfected CCF. Arrows point to transfected CCF. **e,** Expression of the indicated SASP factors in the CCF transfected IMR90 cells was determined by qRT-PCR. Lipo2000 transfection reagent was used as a negative control (n = 4 technically independent experiments). **f-g,** Schematics of Stable Isotope Labeling by Amino acids in Cell culture (SILAC) combined with mass spectrometry analysis used to identify HMGB2-dependent changes in composition of CCF purified from etoposide induced senescent IMR90 cells (**f**). Fold changes of the list of proteins implicated in nucleosome and chromosome-related functionality identified from the analysis in two technical repeats of LC-MS/MS analyses (**g**). Data represent mean ± s.e.m. Scale bar = 20μm. *P* values were calculated using a two-tailed t-test. Source data are provided as a Source Data file.

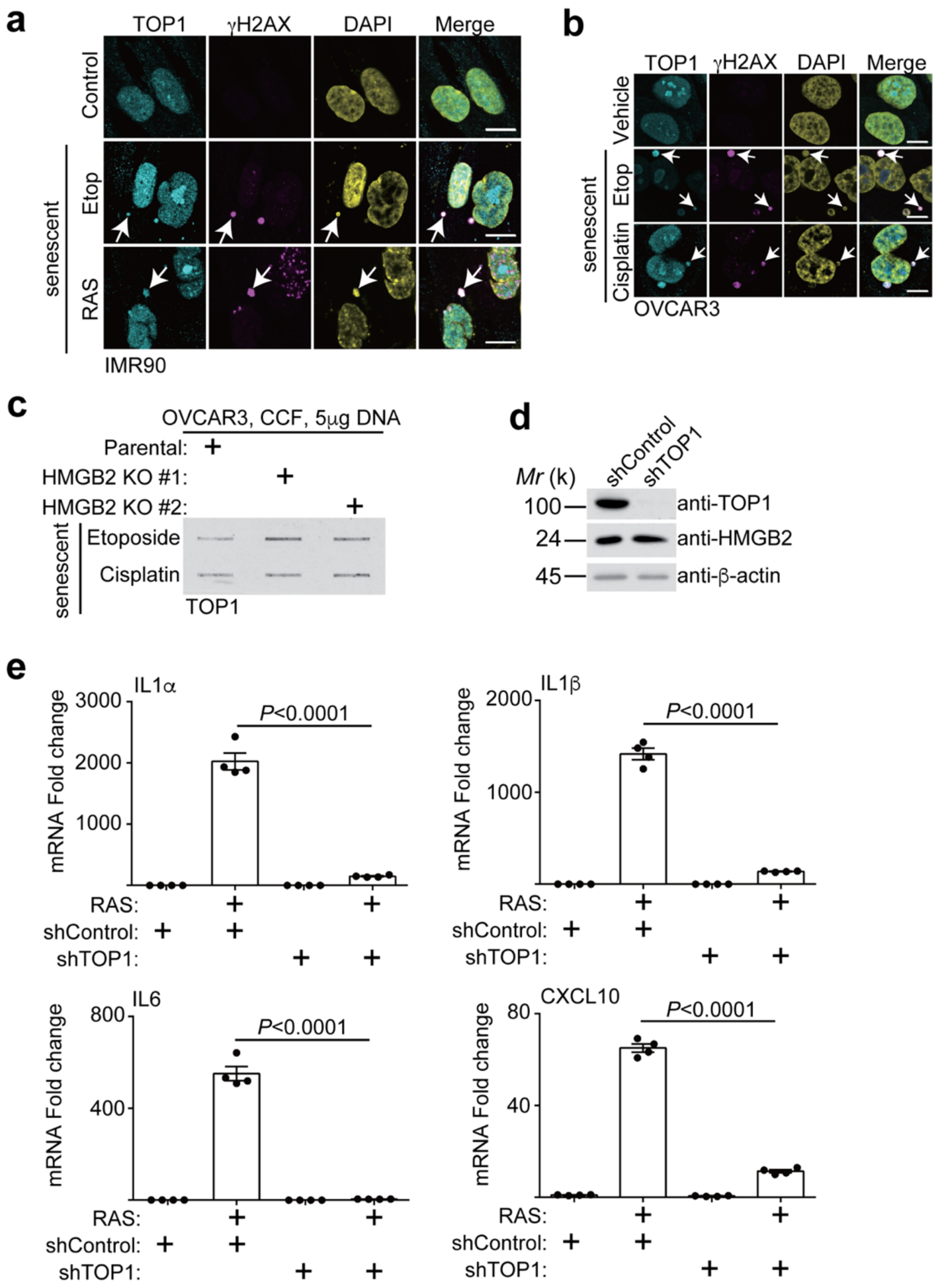

Supplementary Figure 4: TOP1 knockdown suppresses SASP gene expression.
a, Co-staining TOP1 and γH2AX in control and the indicated senescent IMR90 cells. Arrows point to CCF. **b,** Co-staining TOP1 and γH2AX in control and the indicated senescent OVCAR3 cells. Arrows point to CCF. **c,** Slot blot analysis of TOP1 proteins in CCF purified from the indicated senescent OVCAR3 cells with or without HMGB2 knockout. **d-e,** Expression of TOP1, HMGB2 and a loading control β-actin in IMR90 cells expressing shTOP1 or control was determined by immunoblot (**d**). Expression of the indicated SASP factors was determined by qRT-PCR analysis (**e**) (n = 4 biologically independent experiments). Data represent mean ± s.e.m. Scale bar = 10μm. *P* values were calculated using a two-tailed t-test. Source data are provided as a Source Data file.

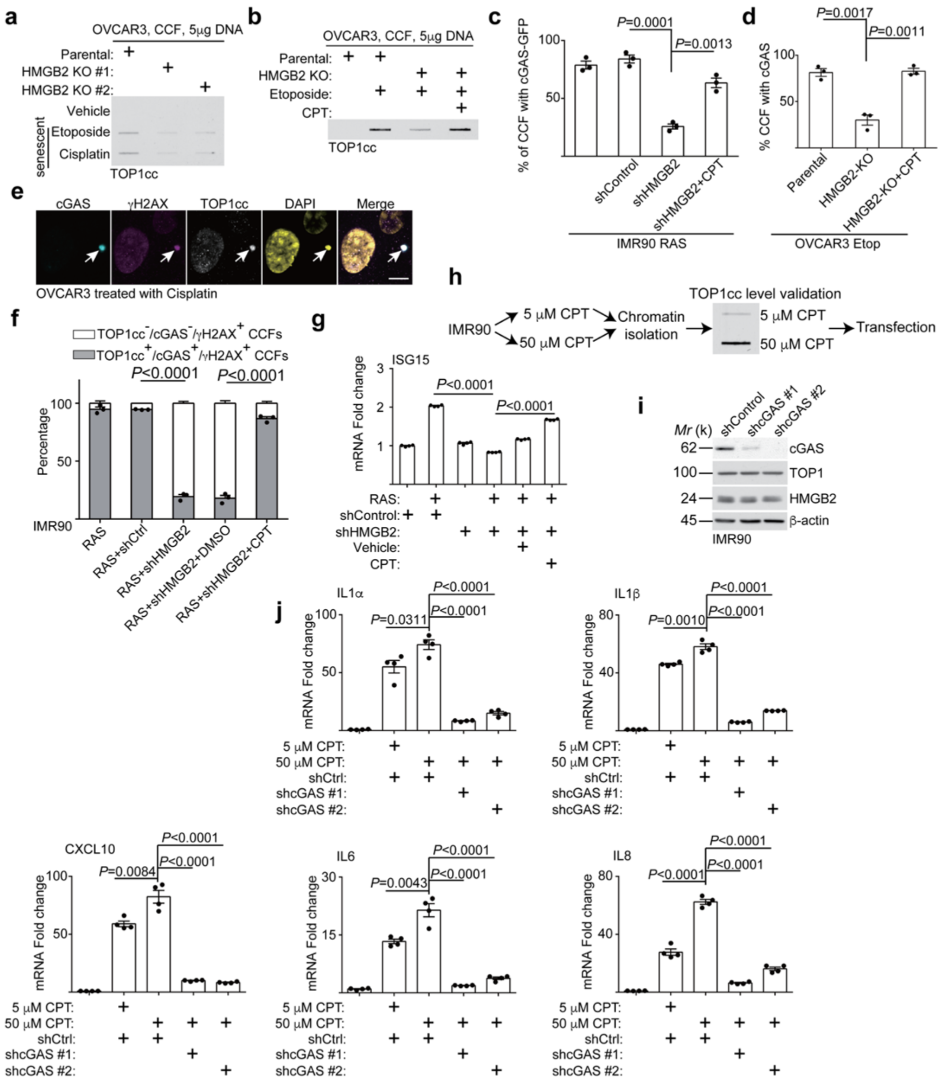

Supplementary Figure 5: TOP1cc is required for cGAS' localization into CCF and SASP gene expression.
a, Slot blot analysis of TOP1cc levels in CCF purified from the indicated cells with or without HMGB2 knockout. **b,** Rescue of the decrease in TOP1cc levels in CCF purified from HMGB2 knockout Etoposide-induced senescent OVCAR3 cells by Camptothecin (CPT) treatment. **c-d,** Quantification of cGAS-GFP (**c**) or endogenous cGAS (**d**) localization into CCF in the indicated senescent cells induced by oncogenic RAS in IMR90 cells or Etoposide treatment in OVCAR3 cells. **e,** Co-staining TOP1cc, cGAS and γH2AX in CCF of the indicated senescent OVCAR3 cells. Arrows point to CCF. Scale bar = 10 μm. **f**, The percentages of the indicated colocalization in the indicated senescent cells. **g**, Expression of *ISG15* in the indicated IMR90 cells determined by qRT-PCR (n = 4 biologically independent experiments). **h,** Schematics of isolation and transfection of CPT-induced TOP1cc positive chromatin fragments. **i-j,** Expression of cGAS, TOP1, HMGB2 and β-actin in IMR90 cells expressing the indicated shcGAS or control was determined by immunoblot (**i**). Expression of the indicated SASP genes was determined by qRT-PCR analysis (n = 4 biologically independent experiments) (**j**). Data represent mean ± s.e.m. n = 3 biologically independent experiments unless otherwise stated. *P* values were calculated using a two-tailed t-test. Source data are provided as a Source Data file.

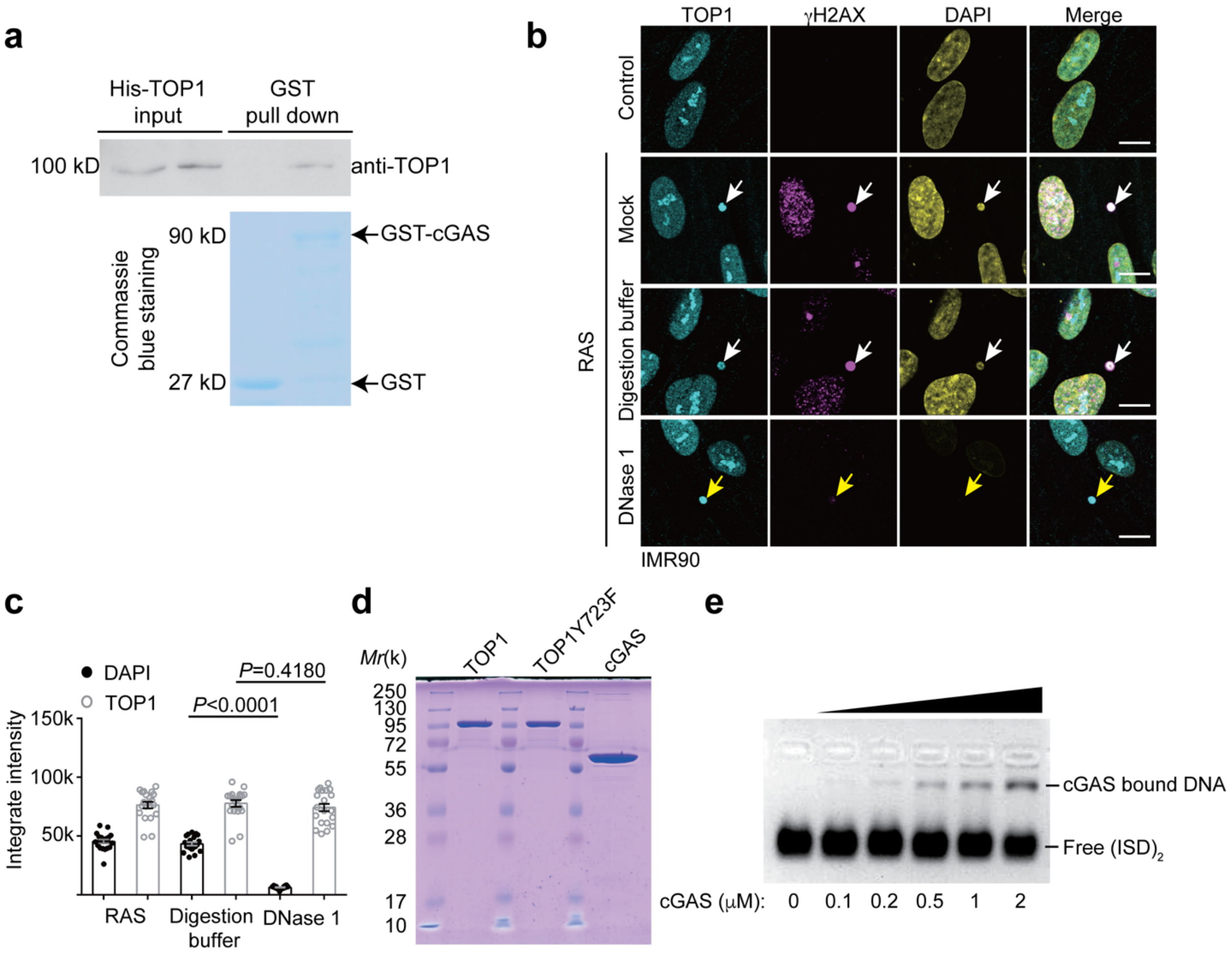

Supplementary Figure 6: Binding of cGAS to dsDNA determined by electrophoretic mobility shift assay.

a, GST pull down assay for the co-incubated purified His-tagged TOP1 using GST or GST-tagged cGAS. The pull down product was subjected to immunoblot analysis using an anti-TOP1 antibody. **b**, Co-staining of TOP1 and γH2AX in CCF in control and the indicated senescent IMR90 cells with or without DNase I digestion. Arrows point to CCF. Scale bar = 10 μm. **c,** The integrated intensity of the indicated markers in CCF of the indicated cells (n = 20 independent CCFs per group). **d,** Coomassie Blue staining the indicated purified proteins used for electrophoretic mobility shift assay. **e,** Dose-dependent dsDNA binding ability of cGAS protein. Source data are provided as a Source Data file.

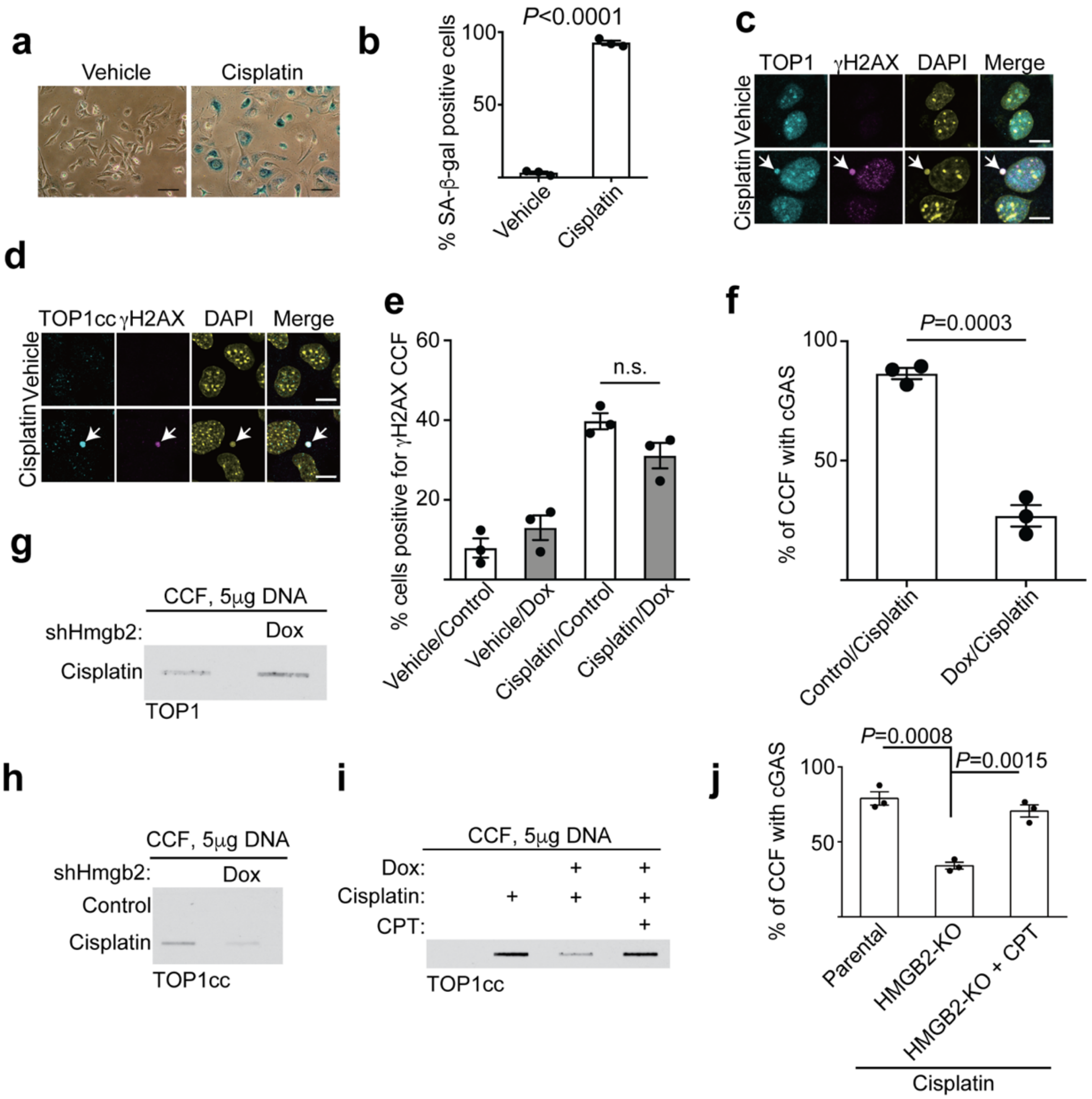

Supplementary Figure 7: HMGB2-TOP1cc-cGAS axis is conserved in cisplatin-induced senescent mouse ID8-*Defb29/Vegf-a* ovarian cancer cells.
a-b, Representative images (**a**) and quantification (**b**) of SA-β-gal staining of ID8-*Defb29/Vegf-a* cells treated without or with Cisplatin to induce senescence. **c-d,** Co-staining of TOP1 (**c**) or TOP1cc (**d**) and γH2AX in CCF of cisplatin-induced senescent ID8-*Defb29/Vegf-a* cells. Arrows point to CCF. **e,** Quantification of γH2AX positive CCF formation in the indicated control and cisplatin-induced senescent ID8-*Defb29/Vegf-a* cells with or without inducible HMGB2 knockdown. **f,** Quantification of cGAS localization into CCF in cisplatin-induced senescent ID8-*Defb29/Vegf-a* cells with or without inducible HMGB2 knockdown. **g-h,** Slot blot analysis of TOP1 (**g**) and TOP1cc (**h**) levels in CCF purified from senescent ID8 cells with or without HMGB2 knockdown. **i,** Rescue of the decrease in TOP1cc levels in CCF purified from HMGB2 knockdown Cisplatin-induced senescent ID8-*Defb29/Vegf-a* cells by Camptothecin (CPT) treatment. **j,** Quantification of cGAS localization into CCF in the indicated Cisplatin-induced senescent ID8-*Defb29/Vegf-a* cells. Data represent mean ± s.e.m. n = 3 biologically independent experiments. Scale bar = 100μm in 7a. Scale bar = 10μm in 7c and 7d. *P* values were calculated using a two-tailed t-test. Source data are provided as a Source Data file.

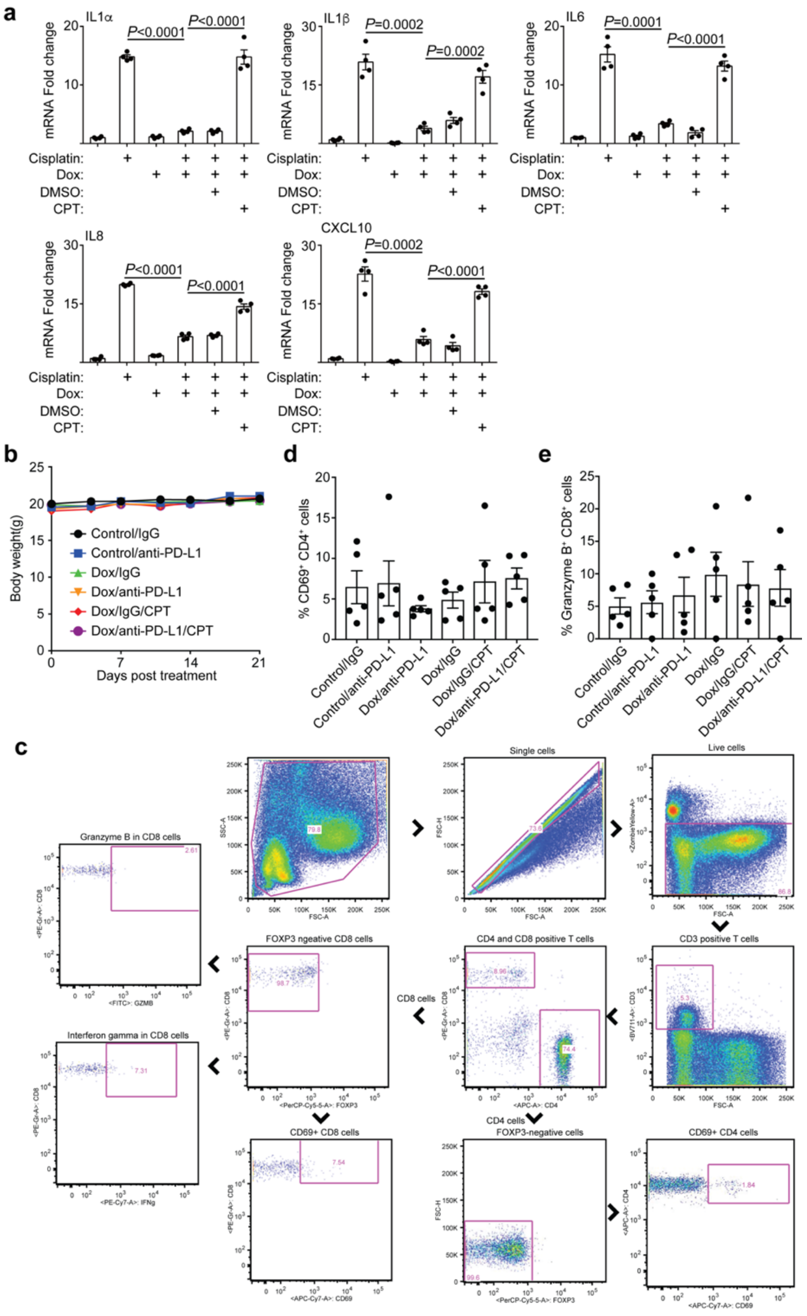

Supplementary Figure 8: anti-PD-L1 and CPT combination does not affect body weight of the tumor bearing mice.

a, Expression of the indicated SASP genes in the indicated ID8-*Defb29/Vegf-a* cells determined by qRT-PCR (n = 4 biologically independent mice per group). **b,** Body weight analysis of mice from the indicated treatment groups during the treatment period (n = 5 biologically independent mice per group). **c**, The gating strategy used for determining the percentages of $CD69^{+}/CD8^{+}$, $IFN\gamma^{+}/CD8^{+}$, $CD69^{+}/CD4^{+}$ and Granzyme $B^{+}/CD8^{+}$ cell populations. **d-e,** At the end of treatment, percentage of CD69 positive CD4 T cells (**d**) and Granzyme B positive CD8 T cells (**e**) was assessed by flow cytometry in the peritoneal wash collected from mice in the indicated treatment groups (n=5 biologically independent mice per group). Data represent mean ± s.e.m. Source data are provided as a Source Data file.
