# Supplementary material for: Topoisomerase 1 cleavage complex enables pattern recognition and inflammation during senescence
Source: Nat Commun. 2020 Feb 19;11:908. doi: 10.1038/s41467-020-14652-y (PMC7031389; doi:10.1038/s41467-020-14652-y)
Supplement: Supplementary file 3 — Reporting Summary [file 41467_2020_14652_MOESM3_ESM.pdf]

nature research

Corresponding author(s): Rugang Zhang

Last updated by author(s): Jan 11, 2020

Reporting Summary

Nature Research wishes to improve the reproducibility of the work that we publish. This form provides structure for consistency and transparency in reporting. For further information on Nature Research policies, see Authors & Referees and the Editorial Policy Checklist.

Statistics

For all statistical analyses, confirm that the following items are present in the figure legend, table legend, main text, or Methods section.

n/a	Confirmed	
☐	☒	The exact sample size (*n*) for each experimental group/condition, given as a discrete number and unit of measurement
☐	☒	A statement on whether measurements were taken from distinct samples or whether the same sample was measured repeatedly
☐	☒	The statistical test(s) used AND whether they are one- or two-sided<br>*Only common tests should be described solely by name; describe more complex techniques in the Methods section.*
☒	☐	A description of all covariates tested
☒	☐	A description of any assumptions or corrections, such as tests of normality and adjustment for multiple comparisons
☒	☐	A full description of the statistical parameters including central tendency (e.g. means) or other basic estimates (e.g. regression coefficient) AND variation (e.g. standard deviation) or associated estimates of uncertainty (e.g. confidence intervals)
☒	☐	For null hypothesis testing, the test statistic (e.g. *F*, *t*, *r*) with confidence intervals, effect sizes, degrees of freedom and *P* value noted<br>*Give P values as exact values whenever suitable.*
☒	☐	For Bayesian analysis, information on the choice of priors and Markov chain Monte Carlo settings
☒	☐	For hierarchical and complex designs, identification of the appropriate level for tests and full reporting of outcomes
☒	☐	Estimates of effect sizes (e.g. Cohen's *d*, Pearson's *r*), indicating how they were calculated

Our web collection on statistics for biologists contains articles on many of the points above.

Software and code

Policy information about availability of computer code

Data collection	NIH Image J was used to quantify slot blot results.
Data analysis	Prism 6 for Mac was used for calculating p values. FlowJo software (TreeStar) or the FlowCore package for FACS analysis. The DAVID bioinformatics resources 6.8 was used for functional classification analysis. Images were analyzed using Live Imaging 4.0 software.

For manuscripts utilizing custom algorithms or software that are central to the research but not yet described in published literature, software must be made available to editors/reviewers. We strongly encourage code deposition in a community repository (e.g. GitHub). See the Nature Research guidelines for submitting code & software for further information.

Data

Policy information about availability of data

All manuscripts must include a data availability statement. This statement should provide the following information, where applicable:

- Accession codes, unique identifiers, or web links for publicly available datasets
- A list of figures that have associated raw data
- A description of any restrictions on data availability

The mass spectrometry proteomics data have been deposited into the MassIVE (http://massive.ucsd.edu) and ProteomeXchange (http://www.proteomexchange.org) data repository with the accession number MSV000084789 and PXD017070, respectively. All the data supporting the findings of this study are available within the article and its supplementary information files and from the corresponding author upon reasonable request. A reporting summary for this article is available as a Supplementary Information file.

Field-specific reporting

Please select the one below that is the best fit for your research. If you are not sure, read the appropriate sections before making your selection.

- [x] Life sciences
- [ ] Behavioural & social sciences
- [ ] Ecological, evolutionary & environmental sciences

For a reference copy of the document with all sections, see nature.com/documents/nr-reporting-summary-flat.pdf

Life sciences study design

All studies must disclose on these points even when the disclosure is negative.

Sample size	Results from in vitro experiments were used to determine the in vivo sample size. At least 5 mice in each experimental groups.
Data exclusions	There was no exclusion from the experiments.
Replication	All attempts at replication were successful.
Randomization	Experiments were all randomized.
Blinding	Analysis was performed blindly.

Reporting for specific materials, systems and methods

We require information from authors about some types of materials, experimental systems and methods used in many studies. Here, indicate whether each material, system or method listed is relevant to your study. If you are not sure if a list item applies to your research, read the appropriate section before selecting a response.

Materials & experimental systems

n/a	Involved in the study	
☐	☒	Antibodies
☐	☒	Eukaryotic cell lines
☒	☐	Palaeontology
☐	☒	Animals and other organisms
☒	☐	Human research participants
☒	☐	Clinical data

Methods

n/a	Involved in the study	
☒	☐	ChIP-seq
☐	☒	Flow cytometry
☒	☐	MRI-based neuroimaging

Antibodies

Antibodies used	The following antibodies were purchased from the indicated suppliers and used for immunoblotting or immunostaining at the indicated concentrations: mouse monoclonal anti-gH2AX (clone JBW301) (Millipore. Cat. No: 05-636), 1:500 for immunofluorescence; rabbit monoclonal anti-gH2AX (20E3) (Cell Signaling Technology. Cat. No: 9718), 1:500 for immunofluorescence; Alexa Fluor® 594 anti-gH2AX (2F3) (Biolegend. Cat. No: 613410), 1:200 for immunofluorescence; rabbit polyclonal anti-HMGB2 (Abcam. Cat. No: 67282), 1:1000 for immunoblotting and 1:500 for immunofluorescence; mouse monoclonal anti-cGAS (D9) (Santa Cruz. Cat. No: sc-515777), 1:200 for immunofluorescence, 1:1000 for immunoblotting; rabbit monoclonal anti-cGAS (D1D3G) (Cell Signaling Technology. Cat. No: 15102), 1:200 for immunofluorescence, 1:1000 for immunoblotting; rabbit monoclonal anti-STING (D2P2F) (Cell Signaling Technology. Cat. No: 13647S), 1:1000 for immunoblotting, rabbit polyclonal anti-Cyclin A (H432) (Santa Cruz. Cat. No: sc-751), 1:1000 for immunoblotting; mouse monoclonal anti-RAS (BD Biosciences. Cat. No: 610001), 1:1000 for immunoblotting; mouse monoclonal anti-P16 (JC8) (Santa Cruz. Cat. No: sc-56330), 1:1000 for immunoblotting; mouse monoclonal anti-P21 (187) (Santa Cruz. Cat. No: sc-817), 1:1000 for immunoblotting; mouse monoclonal anti-[illegible]-actin (Sigma. Cat. No: A2228), 1:10000 for immunoblotting; rabbit polyclonal anti-TOP1 (Proteintech. Cat. No: 20705-1-AP), 1:1000 for immunoblotting and 1:200 for immunofluorescence; mouse monoclonal anti-Topoisomerase I-DNA Covalent Complexes (TOP1cc) (clone 1.1A) (Millipore. Cat. No: MABE1084), 1:1000 for slot blot and 1:200 for immunofluorescence;<br><br>For flow cytometric analysis, APC/CY7 anti-CD69 (Cat. No: 104525), APC anti-CD4 (Cat. No: 100516), PE anti-CD8 (Cat. No: 100708), FITC anti-Granzyme B (Cat. No: 372206), PE/Cy7 anti-interferon-gamma (Cat. No: 505825) antibodies were purchased from Biolegend and used at 1:150 dilutions.
Validation	The antibodies were validated according to the manufacturer.

Eukaryotic cell lines

Policy information about cell lines

Cell line source(s)	IMR90 primary human diploid fibroblasts and human ovarian cancer cell line OVCAR3 were obtained from ATCC, and mouse ovarian cancer cell line ID8-Defb29/Vegf-a was obtained from Dr. Conejo-Garcia
Authentication	Cell lines were re-authenticated by The Wistar Institute's Genomics Facility using short tandem repeat profiling using AmpFLSTR Identifiler PCR Amplification kit (Life Technologies).
Mycoplasma contamination	Regular Mycoplasma testing was performed using LookOut Mycoplasma PCR detection (Sigma).
Commonly misidentified lines (See ICLAC register)	No cell lines used in this study were found in the database of commonly misidentified cell lines that is maintained by ICLAC and NCBI Biosample.

Animals and other organisms

Policy information about studies involving animals; ARRIVE guidelines recommended for reporting animal research

Laboratory animals	C57BL/6 mouse (female, 6–8 weeks old, CRL/NCI)
Wild animals	*Provide details on animals observed in or captured in the field; report species, sex and age where possible. Describe how animals were caught and transported and what happened to captive animals after the study (if killed, explain why and describe method; if released, say where and when) OR state that the study did not involve wild animals.*
Field-collected samples	*For laboratory work with field-collected samples, describe all relevant parameters such as housing, maintenance, temperature, photoperiod and end-of-experiment protocol OR state that the study did not involve samples collected from the field.*
Ethics oversight	The protocols were approved by the Institutional Animal Care and Use Committee of the Wistar Institute

Note that full information on the approval of the study protocol must also be provided in the manuscript.

Flow Cytometry

Plots

Confirm that:

☒ The axis labels state the marker and fluorochrome used (e.g. CD4-FITC).

☒ The axis scales are clearly visible. Include numbers along axes only for bottom left plot of group (a 'group' is an analysis of identical markers).

☒ All plots are contour plots with outliers or pseudocolor plots.

☒ A numerical value for number of cells or percentage (with statistics) is provided.

Methodology

Sample preparation	Mice carrying ID8-Defb29/Vegf cells were sacrificed and peritoneum of each mouse was rinsed using 10ml MACS buffer (1mM EDTA, 2% FBS in PBS). ACK Lysis Buffer (Thermo Fisher, Cat No: A1049201) was used to lyse erythrocytes. Zombie yellow (Biolegend, Cat. No: 423103) was used for viability staining. For extracellular staining fluorophore-conjugated antibodies were incubated at 4 degree for 1 hour. Intracellular staining was performed using eBioscience fixation/permeabilization kit (Thermo Fisher, Cat No: 88-8824-00).
Instrument	LSRII-18 color
Software	DIVA 8.0.1 software was used to collect data, and FlowJo 9.3.2 was used for data analysis
Cell population abundance	*Describe the abundance of the relevant cell populations within post-sort fractions, providing details on the purity of the samples and how it was determined.*
Gating strategy	Cell debris was removed from the analysis using FSC/SSC gate, and doublets were excluded using FSC-A/FSC-H gate. Dead cell were removed using Zombie Yellow viability gate. For cell surface and intracellular stainings were gated as exemplified in the supplementary figure

☒ Tick this box to confirm that a figure exemplifying the gating strategy is provided in the Supplementary Information.
